# Melatonin Modulates Lipid Metabolism in Porcine Cumulus–Oocyte Complex via Its Receptors

**DOI:** 10.3389/fcell.2021.648209

**Published:** 2021-04-01

**Authors:** Tianqi Zhu, Shengyu Guan, Dongying Lv, Mengmeng Zhao, Laiqing Yan, Li Shi, Pengyun Ji, Lu Zhang, Guoshi Liu

**Affiliations:** Key Laboratory of Animal Genetics, Breeding and Reproduction, National Engineering Laboratory for Animal Breeding, Key Laboratory of Animal Genetics Improvement, Ministry of Agriculture, College of Animal Science and Technology, China Agricultural University, Beijing, China

**Keywords:** melatonin, cumulus cell, oocyte, lipid metabolism, melatonin receptor

## Abstract

Lipid is a crucial energy resource for mammalian oocyte. Melatonin could benefit the maturation of porcine oocyte *in vitro*, but the related mechanism is not elucidated yet. In the current study, methods to monitor lipid metabolism in single live oocytes were firstly established using probes (Lipi-Blue and Lipi-Green). It was observed that both lipid biogenesis and lipolysis occurred in maturing oocyte, but the general level of lipids dropped. Then maturing oocytes stained with probes were treated with melatonin or lipid metabolic-related inhibitors (triacsin C, rotenone, or etomoxir). The results showed that the lipid metabolism and maturation of porcine oocytes were all disrupted and that melatonin rescued the oocytes treated with triacsin C or rotenone, but not those treated with etomoxir. Further investigation demonstrated that cumulus cells are able to transfer lipids to oocytes via gap junctions. It was also observed that melatonin receptors exist in cumulus cells and are required for oocytes to maintain lipid metabolism. Meanwhile, the global gene expressing in cumulus cells was also modulated by melatonin, especially the genes related to antioxidants (*SOD1*, *GPX1*, *GPX3*, *GPX4*, *PRDX2*, and *PRDX5*), lipid metabolism (*FABP3*, *FABP5*, *ACACB*, *TECR*, etc.), and mitochondrial respiration (*GPD1*, *ETFB*, *CYC1*, and the genes of ATP synthase). Altogether the current research demonstrates that melatonin modulates lipid metabolism in maturing oocytes through its receptors in cumulus cells and benefits the developmental competence of oocytes.

## Introduction

During its growth phase, as the size of an oocyte increases obviously, a large amount of mRNAs, proteins, and lipids is accumulated in the oocyte cytoplasm. Shortly before ovulation, oocytes progress to meiotic resumption to accomplish the final nuclear and cytoplasmic maturation. These dynamic activities in oocytes consume enormous energy produced from various substrates stored in the oocyte or absorbed from the surroundings, such as glucose, pyruvate, lipids, and amino acids (Dunning et al., 2014). Oocytes that matured in the artificial medium described showed altered metabolism, resulting in lower developmental competence (Bradley and Swann, 2019). Therefore, well-balanced energy substrate provision is definitely required for oocyte maturation and further embryo development, which is critical for both assisted reproductive technology in a clinical setting and embryo production in the livestock industry.

Both glucose and pyruvate can be utilized by porcine oocytes to generate energy for the resumption of meiosis and the acquisition of developmental competence (Lowe et al., 2019). Recently, attention has been placed on the involvement of lipid metabolism in oocyte maturation and preimplantation embryo development. The amount of endogenous lipids in porcine oocytes is exceptionally high compared with that in oocytes of other domestic animals (McEvoy et al., 2000). The reason for such a large amount of intracellular lipid in porcine oocytes and its contribution to energy provision during oocyte maturation and embryo development is still poorly understood.

Porcine oocyte contains about 161 ng lipids, which are much higher than those in mouse (4 ng), sheep (89 ng), and bovine (63 ng) oocytes (McEvoy et al., 2000). Lipids are mainly stored in lipid droplets (LDs), which make porcine oocytes very dark. About 60% of total lipids in porcine oocyte are triglycerides (Sturmey and Leese, 2003). It has been shown that the level of triglycerides decreases as bovine and porcine oocytes progress through meiotic maturation (Ferguson and Leese, 1999; Sturmey and Leese, 2003; Romek et al., 2011), and this process coincides with increased lipolysis (Cetica et al., 2002). The fatty acids (FAs) in mammalian oocytes are cleaved from triglycerides stored in LDs and can be directly transported across the mitochondrial membrane by carnitine palmitoyltransferase (CPT) to be oxidized via β-oxidation (Dunning et al., 2010). The inhibition of CPT in mammalian oocytes by etomoxir (250 μM for mouse, 100 μM for bovine, and 10 μM for porcine) compromises embryo development post fertilization (Paczkowski et al., 2013). Meanwhile, the removal of LDs from mouse metaphase II (MII) oocytes could still develop normally in the blastocyst after *in vitro* fertilization. But blocking the synthesis of lipids by triacsin C, inhibitor of long-chain acyl-CoA synthetases (ACSLs), leads to severe impairment of mouse early embryonic development (Aizawa et al., 2019). Recently, it has been increasingly recognized that lipogenesis and lipolysis are essential for oocyte maturation and embryo development. But the proper status of lipid metabolism in mammalian oocytes is still undetermined because there is no method to monitor and quantify the lipid level in real time.

The beneficial effects of melatonin on the oocyte, sperm, and embryo of animals have been well recognized. It has been determined that melatonin enhances porcine oocyte maturation and embryo development. Firstly, there are about 10^–11^ M melatonin that exist in the follicular fluid of the pig ovary (Shi et al., 2009). It was observed that melatonin appearing in the culture medium could promote oocyte quality and developmental competence (Kang et al., 2009; Shi et al., 2009). The expression of the melatonin receptor 1 (*MT1*) gene was identified in cumulus and granulosa cells, but not in oocytes (Kang et al., 2009). Meanwhile, numerous researches had been conducted to investigate the role of melatonin on mammalian reproductive activities. The results demonstrated that melatonin could protect porcine oocytes from defects induced by heat stress (Li et al., 2015, 2016) or chemical regents such as rotenone, malathion, aflatoxin B1, bisphenol A, benzo(a)pyrene, and MEHP during its maturation *in vitro* (Miao et al., 2018; Park et al., 2018; Zhang et al., 2018; Cheng et al., 2019; Chen et al., 2020; Niu et al., 2020). Melatonin can also enhance mitochondrial biogenesis and reduce endoplasmic reticulum stress during *in vitro* maturation (IVM). Up to now, it is still undetermined whether the beneficial effect of melatonin on porcine oocyte maturation is mediated by its receptors or its direct anti-oxidation capability.

Recently, it has been demonstrated that melatonin regulates lipid metabolism in porcine oocytes, including lipogenesis, lipolysis, and mitochondrial biogenesis. Melatonin treatment significantly elevated the number of LDs and upregulated gene expression related to lipogenesis (Jin et al., 2017). Oocytes treated with melatonin formed smaller LDs and abundantly expressed several genes associated with lipolysis (Jin et al., 2017). Moreover, melatonin significantly increased the content of FAs, mitochondrial numbers, and ATP as indicated by fluorescent staining (Jin et al., 2017). Concomitantly, melatonin treatment upregulated gene expression related to FA β-oxidation and mitochondrial biogenesis (Jin et al., 2017). These results indicated that melatonin promoted lipid metabolism and thereby provided an energy source for oocyte maturation and subsequent embryonic development. However, another study showed that melatonin added to an IVM medium enhances LD accumulation as well as triglyceride level in porcine oocytes (He et al., 2018). Melatonin supplementation decreased mitochondrial membrane potential, mitochondrial respiratory chain complex IV activity, and mitochondrial reactive oxygen species in maturing oocytes, which indicated that melatonin suppresses mitochondrial activities in porcine oocytes (He et al., 2018). Further investigation found that melatonin did not alter the copy number of mitochondrial DNA (mtDNA) but reduced the expression of mtDNA-encoded genes, which is mediated by DNMT1 (He et al., 2018). These results suggest that melatonin promotes oocyte maturation by inducing mitochondrial quiescence to reduce mROS production and enhance LD accumulation in porcine oocytes (He et al., 2018).

The evidence from the above studies demonstrated that melatonin promotes porcine oocyte maturation by regulating lipid metabolism. But it is still unrevealed whether melatonin affects lipid biogenesis or lipolysis in porcine oocytes. In the current study, the methods for monitoring lipid dynamics in maturing porcine oocytes were established using specific probes. Then the regulation of melatonin on lipid metabolism in porcine oocytes was evaluated. Meanwhile, the role of cumulus cells and their melatonin receptors in modulating lipid contents in oocyte was also evaluated by inhibiting gap junctions and melatonin receptors. The current results showed that melatonin enhances porcine oocyte maturation by regulating lipid transfer from cumulus cells to oocytes via melatonin receptors.

## Materials and Methods

### Ethics Statement

All the procedures for animal manipulations were performed according to the guidelines of the Animal Care and Use Committee of China Agricultural University and approved by the Ethics Committee of the Agriculture University of China (permission number: CAU20150915-1 SYXK).

### Chemicals

All chemicals used in this study were purchased from the Sigma-Aldrich Chemical Company (St. Louis, MO, United States) unless otherwise indicated.

### Oocyte IVM

Ovaries collected from pre-pubertal gilts were donated by a local slaughterhouse (Beijing Food Company, Beijing, China). Follicular fluid was aspirated from follicles (3–6 mm in diameter) using a syringe and a 20-G hypodermic needle. The cumulus–oocyte complexes (COCs) were rinsed twice in HEPES-buffered Tyrode’s Lactate (TL-HEPES) medium and then three times in maturation medium without hormones. The COCs were then transferred into the maturation medium (50 oocytes per 0.5 ml of medium) consisting of TCM-199 supplemented with 0.57 mM cysteine, 3.05 mM D-glucose, 0.91 mM sodium pyruvate, 10 ng/ml epidermal growth factor (EGF), 0.5 IU/ml ovine luteinizing hormone (LH), 0.5 IU/ml porcine follicle-stimulating hormone (FSH), 0.1% polyvinyl alcohol (PVA), 75 mg/ml penicillin, 50 mg/ml streptomycin, 20 ng/ml LIF (EMD Millipore, MA, United States), 20 ng/ml IGF1 (ProSpec, Ness Ziona, Israel), and 40 ng/ml FGF2 (PeproTech, NJ, United States) (Abeydeera et al., 1998; Yuan et al., 2017). Meanwhile, the inhibitors or dyes applied to study lipid metabolism were added to the maturation medium as described in each paragraph. Three inhibitors were used to study lipid metabolism as described in the literature. Triacsin C served as an inhibitor of ACSLs, could block the synthesis of lipids, and could induce severe impairment of mouse early embryonic development (Aizawa et al., 2019). Etomoxir could irreversibly inhibit FA oxidation (FAO) by blocking CPT-1a (Lowe et al., 2019). Rotenone could disrupt the mitochondrial electron transport chain (ETC) in maturing oocytes through the inhibition of the ETC complex I, resulting in ATP depletion, mROS production, and damage of mitochondrial membrane potential (Niu et al., 2020). Maturation was conducted at 38.5°C and 5% CO_2_ in air with 100% humidity. After 42–44 h of maturation, the COCs were transferred into TL-HEPES containing 1 mg/ml hyaluronidase, and the cumulus cells were removed by vortex. The oocytes and cumulus cells were rinsed with TL-HEPES and then used for subsequent experiments.

### Parthenogenetic Activation of Oocytes

The denuded porcine oocytes were activated in the activation medium [0.3 m mannitol, 0.05 mm CaCl_2_, 0.1 mm MgCl_2_, and 0.1% bovine serum albumin (BSA)] by an electrical pulse of DC 130 V/mm for 80 μs using a BTX Electro-Cell Manipulator 2001 (BTX, Inc., San Diego, CA, United States). The oocytes were then rinsed in porcine zygote medium-3 (PZM-3) and cultured in the medium containing 5 μg/ml of cytochalasin B for 5–6 h, at 38.5°C and 5% CO_2_ in air with 100% humidity. Subsequently, oocytes were rinsed in PZM supplemented with 0.6 mg/ml of FA-free BSA for embryo culture.

### *In vitro* Culture (IVC) of Embryos

Parthenogenetically activated oocytes, in groups of approximately 15–20, were cultured in 100 μl droplets of PZM-3 supplemented with 0.6 mg/ml of BSA. Melatonin stock dissolved in TCM-199 was added to the culture medium according to the experimental design. The oocytes were then cultured in the medium at 39°C, 5% CO_2_, and 5% O_2_. The cleavage rate and blastocyst rate were observed and recorded at 48 and 168 h of IVC, respectively.

### Observation of LDs in Porcine and Mouse Oocytes

The COCs were placed in the TL-HEPES medium containing 0.1 μmol/L Lipi-Blue (Dojindo Laboratories, Kumamoto, Japan) for 30 min and then incubated in maturation medium. At 18 and 44 h of maturation, the COCs were taken out from the IVM medium to be stained by Lipi-Green (Dojindo Laboratories, Kumamoto, Japan). The cumulus cells surrounding oocytes were removed, and then the MII oocytes were placed in a glass petri dish for imaging using a confocal microscope (Nikon A1 HD25, Tokyo, Japan) with excitation wavelength λex = 405 nm for Lipi-Blue and λex = 488 nm for Lipi-Green. ImageJ (Version 1.53, National Institutes of Health, United States) was used to process the images and record the fluorescence intensities of LDs.

Mouse oocytes from melatonin receptor-knockout (*Mtnr1a*^–/–^ and *Mtnr1b*^–/–^) or wild-type mice were collected as described in our previous research (Zhang et al., 2019). Briefly, at the age of 8 weeks, the mice were injected with 5 IU PMSG, 46–48 h later, followed by 5 IU hCG to induce superovulation. Thirteen to fifteen hours after hCG injection, COCs at MII stage were isolated from female mice’s ampullas. Then denuded MII oocytes were stained with 0.1 μmol/L of Lipi-Blue for 30 min, and then the mouse oocytes were placed in a glass petri dish for imaging with a confocal microscope.

### Lipid Analysis by UPLC–MS/MS

#### Lipid Extraction

Denuded MII oocytes were washed three times with phosphate-buffered saline (PBS) solution and stored in microtubes at −80°C until analysis. The extraction was performed according to the protocol described by Mateus et al. (2016) with modifications (Sudano et al., 2016). Four hundred oocytes per sample were counted, and the metabolites were extracted using a solution of 400 μl methanol:water (4:1, v/v). The mixture was settled at −20°C and treated by a high-throughput tissue crusher Wonbio-96c (Shanghai Wanbo Biotechnology Co., Ltd.) at 50 Hz for 6 min; then the samples were placed at −20°C for 30 min to precipitate proteins. After centrifugation at 13,000 g at 4°C for 15 min, the supernatant was carefully transferred to sample vials for LC–MS/MS analysis.

#### UPLC–MS/MS Analysis

Chromatographic separation of the metabolites was performed on an ExionLC^TM^ AD system (AB Sciex, United States) equipped with an ACQUITY UPLC BEH C18 column (100 mm × 2.1 mm i.d., 1.7 μm; Waters, Milford, United States). The mobile phases consisted of 0.1% formic acid in water (solvent A) and 0.1% formic acid in acetonitrile:isopropanol (1:1, v/v) (solvent B). The solvent gradient changed according to the following conditions: from 0 to 3 min, 95% (A):5% (B) to 80% (A):20% (B); from 3 to 9 min, 80% (A):20% (B) to 5% (A):95% (B); from 9 to 13 min, 5% (A):95% (B) to 5% (A):95% (B); from 13 to 13.1 min, 5% (A):95% (B) to 95% (A):5% (B); and from 13.1 to 16 min, 95% (A):5% (B) to 95% (A):5% (B) to equilibrate the systems. The column temperature was maintained at 40°C. The sample injection volume was 20 μl, and the flow rate was set to 0.4 ml/min. The UPLC system was coupled to a quadrupole-time-of-flight mass spectrometer (Triple TOF^TM^ 5600^+^, AB Sciex, United States) equipped with an electrospray ionization (ESI) source operating in positive and negative modes. Data acquisition was performed with the data-dependent acquisition (DDA) mode. The detection was carried out over a mass range of 50–1,000 *m*/*z*.

#### Data Preprocessing and Annotation

After UPLC--TOF/MS analyses, the raw data were processed by Progenesis QI 2.3 (Non-linear Dynamics, Waters, United States) for peak detection and alignment. Metabolic features that detected at least 80% in any set of samples were retained. Mass spectra of the metabolic features were identified by using the accurate mass, MS/MS fragments spectra, and isotope ratio difference by searching in reliable biochemical databases such as the Human Metabolome Database (HMDB)^1^ and METLIN database^2^. Concretely, the mass tolerance between the measured *m*/*z* values and the exact mass of the components of interest was ±10 ppm. For metabolites having MS/MS confirmation, only the ones with an MS/MS fragment score above 30 were considered as confidently identified. Otherwise, metabolites had only tentative assignments.

### Transcriptomic Analysis of Cumulus Cells

#### RNA Extraction

The cumulus cells were collected from COCs (*n* = 300 oocytes, six replicates), and then the total RNA was extracted from the tissue using the TRIzol reagent according to the manufacturer’s instructions (Invitrogen), and genomic DNA was removed using DNase I (Takara). Then RNA quality was determined by the 2100 Bioanalyzer (Agilent) and quantified using the ND-2000 (NanoDrop Technologies). Only samples with high-quality RNA (OD260/280 = 1.8–2.2, OD260/230 ≥ 2.0, RIN ≥ 6.5, 28S:18S ≥ 1.0, > 2 μg) were selected to construct the sequencing library.

#### Library Preparation and Sequencing

The RNA-seq transcriptome library was prepared following a TruSeq^TM^ RNA sample preparation kit from Illumina (San Diego, CA) using 1 μg of total RNA. Libraries were selected for cDNA target fragments of 200–300 bp on 2% low-range ultra-agarose followed by PCR amplified using Phusion DNA polymerase (NEB) for 15 PCR cycles. After being quantified by TBS380, the paired-end RNA-seq sequencing library was sequenced with the Illumina HiSeq X Ten/NovaSeq 6000 sequencer (San Diego, CA).

#### Differential Expression Analysis and Functional Enrichment

The raw paired-end reads were trimmed and quality controlled by SeqPrep^3^ and Sickle^4^ with default parameters. Then clean reads were separately aligned to the reference genome^5^ with an orientation mode using TopHat^6^ (version 2.0.0) software (Trapnell et al., 2009).

To identify differentially expressed genes (DEGs) between two different samples, the expression level of each transcript was calculated according to the fragments per kilobase of exon per million mapped reads (FRKM) method. RSEM^7^ was used to quantify gene abundances (Li and Dewey, 2011). R statistical package software EdgeR^8^ (Robinson et al., 2010) was utilized for differential expression analysis. In addition, functional enrichment analyses including GO and KEGG were performed to identify which DEGs were significantly enriched in GO terms and metabolic pathways at a Bonferroni-corrected *P* < 0.05 compared with the whole-transcriptome background. GO functional enrichment and KEGG pathway analysis were carried out with Goatools^9^ and KOBAS^10^ (Xie et al., 2011).

### Gene Expression by Quantitative Real-Time PCR (RT-PCR)

Cumulus cells were collected from COCs and then washed three times with PBS and stored at −80°C until RNA extraction. Total RNA was extracted using TRIzol (Invitrogen Inc., Carlsbad, CA, United States) and quantified by measuring absorbance at 260 nm and stored at −80°C until assay. The mRNA levels of relevant genes were evaluated by quantitative RT-PCR using the One-Step SYBR PrimeScript RT-PCR Kit (Takara Bio. Inc., Tokyo, Japan) in a LightCycler (Roche Applied Science, Mannheim, Germany). Accumulated levels of fluorescence were analyzed by the second derivative method after the melting curve analysis, and then the expression levels of target genes were normalized to the expression level of GAPDH in each sample. Primer pairs of analyzed mRNAs are described in Table 1.

**TABLE 1:** The primers used for real-time q-PCR.

Target genes	Accession number	Primer sequences (5′–3′)	
GAPDH	NM_001206359.1	F	GTCGGTTGTGGATCTGACCT
		R	GTCCTCAGTGTAGCCCAGGA
ACACA	NM_001114269.1	F	TTTGTTACTCGTTTTGGTGGGA
		R	AGCGTTGGCTTTCAGGTCTT
FASN	NM_001099930.1	F	TGTCCTGGGAAGAGTGTAAGCA
		R	GCAGGAACTCGGACATAGCG
PLIN2	NM_214200.2	F	ATGGCTGGCGACATCTACTCA
		R	TGCCCCTTGCTGGAACTG
CGI-58	NM_001012407.1	F	AGGAGGTCTCGGACTTTGGG
		R	GGTCTGGTCGCTCAGGAAAA
PPARγ	NM_214379.1	F	GCATCTTTCAGGGGTGTCAGTT
		R	CGTGGACGCCATACTTTAGGA
CPT2	NM_001246243.1	F	TGTCCCAGTATTTTCGGCTTTT
		R	GTCTCCTCGTTGCCACCCT
CPT1a	NM_001129805.1	F	TAAACGGATGACGGCTCTGG
		R	TGTGGGTCGGGGTGATGT
PGC-1α	NM_213963.2	F	AATCGCAGTCGCAACATTTACA
		R	TGGGTCCCCGAAGACTCAC
TFAM	NM_001130211.1	F	GGACCTCTGTGCGGTTTGTG
		R	CACCTGCCAGTCTGCCCTA
PRDX2	NM_001244474.1	F	TGGGACGCTCTGTGGATGA
		R	GGGGCAGGTCTGGCTTTT

### Statistical Analyses

Unless specifically described, the rest of the data were expressed as mean ± SEM. The significance of differences between mean values was analyzed by ANOVA, followed by Dunnett’s *post hoc* test using SPSS 18.0 statistical software (SPSS Inc., Chicago, IL, United States). The significant difference between treatments was set to *P* < 0.05.

For lipidomics analysis of porcine oocyte, multivariate statistical analysis was performed using the ropls R package (Version 1.6.2)^11^. Principal component analysis (PCA) using an unsupervised method was applied to obtain an overview of the metabolic data, general clustering, trends, or outliers, which were visualized. Variable importance (VIP) was calculated in the OPLS-DA model. *P* values were estimated with paired Student’s *t*-test on single-dimensional statistical analysis. Statistical significance among groups was considered as a VIP value more than 1 and *P* < 0.05.

## Results

### Dynamics of LDs in Porcine Oocyte During IVM

**FIGURE 1:**
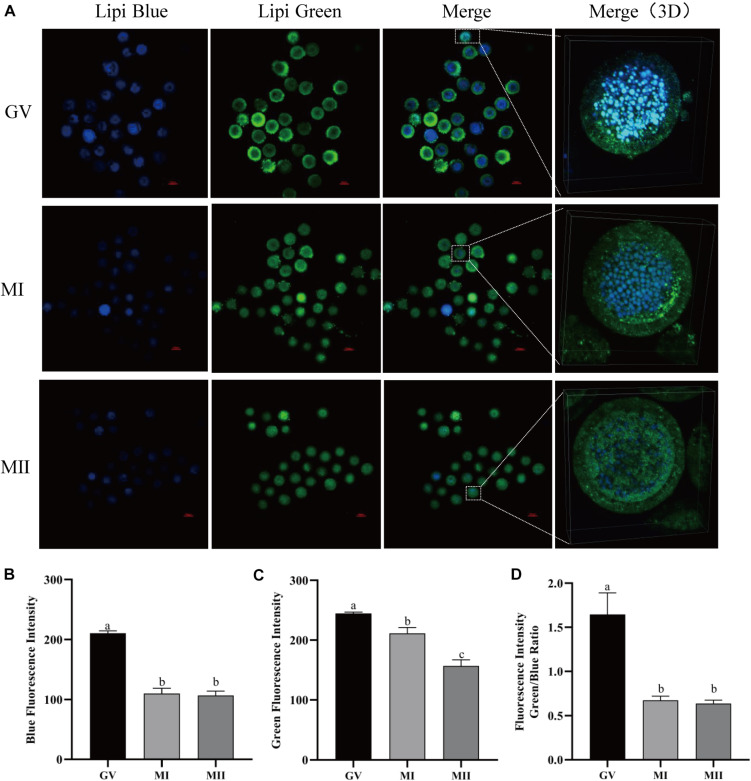
The dynamics of LDs in pig oocytes during *in vitro* maturation. **(A)** Representative images of Lipi-Blue fluorescence (blue) and Lipi-Green fluorescence (green) in germinal vesicle (GV)-, MI-, and MII-stage oocytes. Scale bar: 100 μm. **(B)** Quantification of fluorescence intensity of Lipi-Blue in GV- (*n* = 31), MI- (*n* = 41), and MII-stage (*n* = 44) oocytes. **(C)** Quantification of fluorescence intensity of Lipi-Green in GV- (*n* = 30), MI- (*n* = 37), and MII-stage (*n* = 43) oocytes. **(D)** Ratio of fluorescence intensity of Lipi-Blue and Lipi-Green in GV- (*n* = 45), MI- (*n* = 64), and MII-stage (*n* = 56) oocytes. Student’s *t*-test and one-way ANOVA were utilized for statistical analyses. Different lowercase letters indicate significant differences. Error bars indicate SEM.

In order to detect the LDs in living oocytes, lipid-specific probes (Lipi-Blue and Lipi-Green) were tested in porcine oocytes at different maturation phases. As described by a previous study, both probes can be used to monitor the lipid changes in living cells for over 48 h (Tatenaka et al., 2019). It was observed that both blue and green fluorescence decreased as the oocyte grew from the GV to MII stages (Figure 1A). The oocytes were stained with Lipi-Blue at the GV stage, and then the images were acquired at the GV (210.52 ± 4.03), MI (110.06 ± 8.92), and MII (106.87 ± 7.34) stages, so the decrease of blue fluorescence intensity indicates the mobilization of lipids to generate energy for the activities of oocytes (Figure 1B). Meanwhile, the oocytes were co-incubated with Lipi-Green at the GV, MI, and MII stages; thus, the intensity of green fluorescence represents the total lipids contained in each time point, which are also reduced as oocytes grow (Figure 1C) from stages GV (244.75 ± 2.25) to MI (211.26 ± 9.85) to MII (156.80 ± 10.44). The ratio of Lipi-Green/Lipi-Blue is also lower in the MII oocytes (Figure 1D) (GV: 1.645 ± 0.25, MI: 0.67 ± 0.047, and MII: 0.64 ± 0.038). These observations provide a method to monitor the dynamics of lipid in single living oocytes.

### Melatonin Modulates the Lipid Metabolism in Porcine Oocyte

**FIGURE 2:**
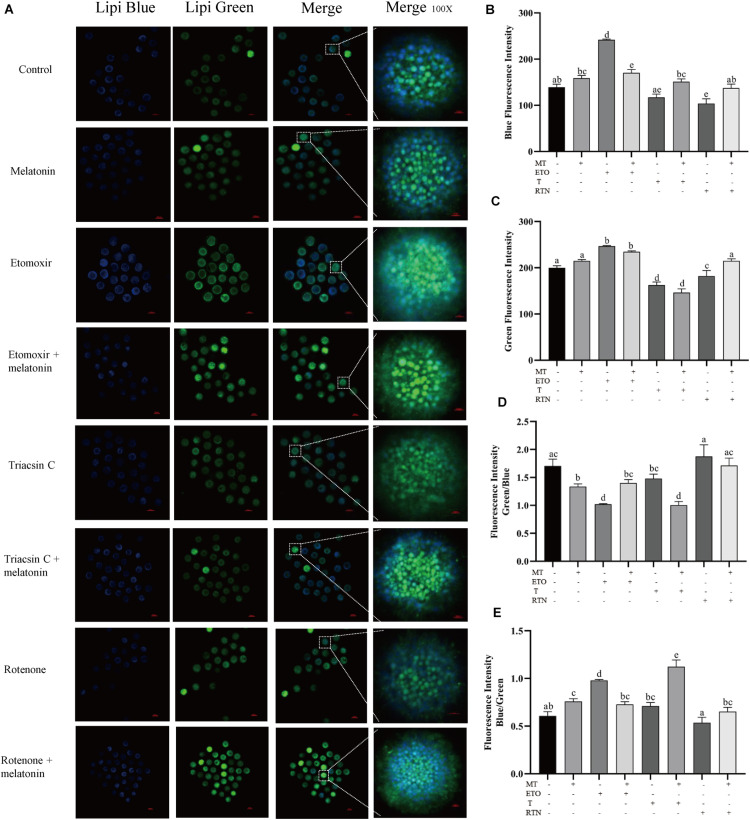
Melatonin modulates lipid metabolism in oocytes. **(A)** Representative images of Lipi-Blue fluorescence (blue) and Lipi-Green fluorescence (green) in porcine oocytes stained in GV stage but pictured in MII stage. Scale bar: 100 μm. **(B–E)** Quantification of fluorescence intensity of Lipi-Blue and Lipi-Green and the ratio of Lipi-Blue/Lipi-Green or Lipi-Green/Lipi-Blue in porcine oocytes treated with control (*n* = 57), melatonin (*n* = 27), etomoxir (*n* = 21), etomoxir + melatonin (*n* = 25), triacsin C (*n* = 27), triacsin C + melatonin (*n* = 30), rotenone (*n* = 19), and rotenone + melatonin (*n* = 35). Student’s *t*-test and one-way ANOVA were utilized for statistical analyses. Different lowercase letters indicate significant differences. Error bars indicate SEM.

Agonists targeted at the pathway of lipid metabolism were applied to investigate how melatonin regulates lipid metabolism in maturing porcine oocytes (Figure 2). Compared with the control group (139.11 ± 6.58), melatonin-treated oocytes showed higher blue fluorescence intensity (158.77 ± 5.96), which indicates that less lipids stored in GV oocytes were mobilized. When the oocytes were treated with etomoxir, an irreversible inhibitor of FAO blocking CPT-1a (Lowe et al., 2019), the blue and green fluorescence intensities (241.83 ± 1.64) are all significantly higher than that in oocytes of the control group, and melatonin cannot reverse these results.

When the lipogenesis of oocytes was suppressed by triacsin C, an inhibitor of ACSLs which can block the *de novo* synthesis of triglycerides (Aizawa et al., 2019), diglycerides, and cholesterol esters, the blue fluorescence intensity (117.32 ± 6.85) is significantly lower than that in oocytes of the control group, and melatonin treatment could reverse this result (151.11 ± 6.10). Similarly, the disruption of the mitochondrial ETC in maturing oocytes through the inhibition of complex I by rotenone (Won et al., 2015) leads to reduced blue fluorescence intensity (103.85 ± 10.26), which was reversed by melatonin (137.39 ± 8.43). Altogether, these results indicate that melatonin suppressed lipolysis to generate energy and promote lipid store in maturing porcine oocytes.

To detect the changes of lipid contents induced by melatonin, 400 oocytes were collected for each sample with seven repeats for both the melatonin and control groups and were used for the UPLC–MS/MS study. In total, 298 FAs were identified. As the results showed in Table 2, the levels of 13 lipids were altered by melatonin; among them, eight FAs were upregulated, and five FAs were downregulated.

**TABLE 2:** The fatty acid profiles of oocytes from the control and melatonin groups.

Metabolites	Mz	Type	IonFormula	Class	Subclass	FC(MT/CON)	*P*-value
Cer(d18:1/16:0)	538.5194	Cer(d18:1/16:0) + H	C34 H68 O3 N1	SP	Cer	0.713011332	0.020166
MG(20:1p)	386.3629	MG(20:1p) + NH4	C23 H48 O3 N1	GL	MG	4.056503024	0.020551
PC(34:0)	762.6007	PC(34:0) + H	C42 H85 O8 N1 P1	GP	PC	0.613003343	0.035028
PC(34:3)	756.5538	PC(34:3) + H	C42 H79 O8 N1 P1	GP	PC	1.7938854	0.041914
PC(38:5)	808.5851	PC(38:5) + H	C46 H83 O8 N1 P1	GP	PC	0.555480605	0.018347
PE(20:0p)	530.3217	PE(20:0p) + Na	C25 H50 O7 N1 P1 Na1	GP	PE	1.32368186	0.019753
TG(8:0/8:0/8:0)	488.3946	TG(8:0/8:0/8:0) + NH4	C27 H54 O6 N1	GL	TG	1.814711539	0.047532
TG(16:0/18:1/18:1)	876.8015	TG(16:0/18:1/18:1) + NH4	C55 H106 O6 N1	GL	TG	5.550013905	0.036007
LPE(22:3)	530.3252	LPE(22:3)-H	C27 H49 O7 N1 P1	GP	LPE	1.456611091	0.012236
LPE(22:4)	528.3096	LPE(22:4)-H	C27 H47 O7 N1 P1	GP	LPE	1.356583578	0.009065
LPI(18:0)	599.3202	LPI(18:0)-H	C27 H52 O12 N0 P1	GP	LPI	1.859655426	0.038688
SM(d36:1)	775.5971	SM(d36:1) + HCOO	C42 H84 O8 N2 P1	SP	SM	0.616533814	0.025273
PC(37:4)	796.5851	PC(37:4) + H	C45 H83 O8 N1 P1	GP	PC	0.195726296	0.022136

### Lipid Metabolism Regulates the Developmental Competence of Porcine Oocytes

**FIGURE 3:**
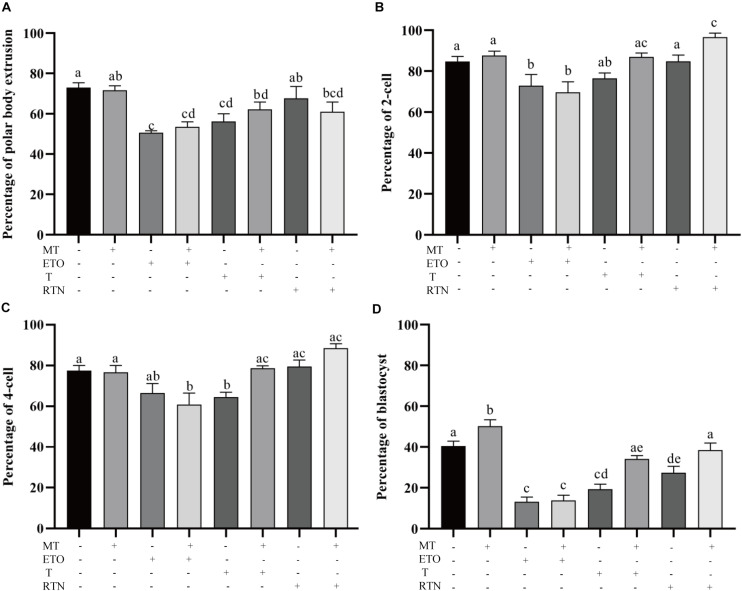
Lipid metabolism regulates the developmental competence of porcine oocytes. **(A)** The percentage of polar body extrusion in porcine oocytes treated with control (*n* = 2,386), melatonin (*n* = 894), etomoxir (*n* = 1,303), etomoxir + melatonin (*n* = 1,301), triacsin C (*n* = 931), triacsin C + melatonin (*n* = 1,026), rotenone (*n* = 710), and rotenone + melatonin (*n* = 706). **(B)** The percentage of two-cell embryos in porcine oocytes treated with control (*n* = 899), melatonin (*n* = 445), etomoxir (*n* = 739), etomoxir + melatonin (*n* = 654), triacsin C (*n* = 207), triacsin C + melatonin (*n* = 315), rotenone (*n* = 414), and rotenone + melatonin (*n* = 328). **(C)** The percentage of four-cell embryos in porcine oocytes treated with control (*n* = 899), melatonin (*n* = 445), etomoxir (*n* = 739), etomoxir + melatonin (*n* = 654), triacsin C (*n* = 207), triacsin C + melatonin (*n* = 315), rotenone (*n* = 414), and rotenone + melatonin (*n* = 328). **(D)** The percentage of blastocysts in porcine oocytes treated with control (*n* = 899), melatonin (*n* = 445), etomoxir (*n* = 739), etomoxir + melatonin (*n* = 654), triacsin C (*n* = 207), triacsin C + melatonin (*n* = 315), rotenone (*n* = 414), and rotenone + melatonin (*n* = 328). Student’s *t*-test and one-way ANOVA were utilized for statistical analyses. Different lowercase letters indicate significant differences. Error bars indicate SEM.

To determine how melatonin affects the maturation of porcine oocytes through modulating the lipid metabolism, the maturing oocytes were treated with the agonists used above (Figure 3). Firstly, the supplementation of melatonin does not alter the first polar body extrusion (71.63 ± 2.26% vs. 72.94 ± 2.475%), which represents nuclear maturation, but rescues the defects caused by triacsin C (56.19 ± 38.6% vs. 62.17 ± 3.6%) and rotenone (67.60 ± 5.9% vs. 61.05 ± 4.8%) (Figure 3A). Melatonin is unable to reverse the effect of etomoxir (50.63 ± 1.03% vs. 53.57 ± 2.4%). Those oocytes were then activated parthenogenetically and cultured for further development. As shown in Figures 3B–D, melatonin promotes the embryo development indicated by the higher rate of blastocyst formation (48.01 ± 3.6% vs. 40.42 ± 2.4). In contrast, the treatment of oocytes with etomoxir showed reduced two-cell embryo (72.90 ± 5.494%), four-cell embryo (66.47 ± 4.673%), and blastocyst (13.22 ± 0.02.2%) formation rates, and melatonin could not correct these outcomes. Triacsin C-treated oocytes showed decreased four-cell embryo (64.56 ± 2.289%) and blastocyst (19.28 ± 2.48%) formation rates, which can be rescued by melatonin (78.67 ± 1.2% and 34.12 ± 1.7%, respectively). Oocyte maturing in the medium containing rotenone showed a lower blastocyst formation rate (27.40 ± 3.1%), which can be corrected by melatonin (38.46 ± 0.3.5%). The results presented here demonstrate that lipid metabolism is essential for porcine oocytes to acquire developmental competence and that melatonin suppresses lipid lyse and promotes lipid genesis, leading to enhanced developmental competence.

### Cumulus Cells Participate in the Regulation of Lipid Metabolism in Porcine Oocytes

**FIGURE 4:**
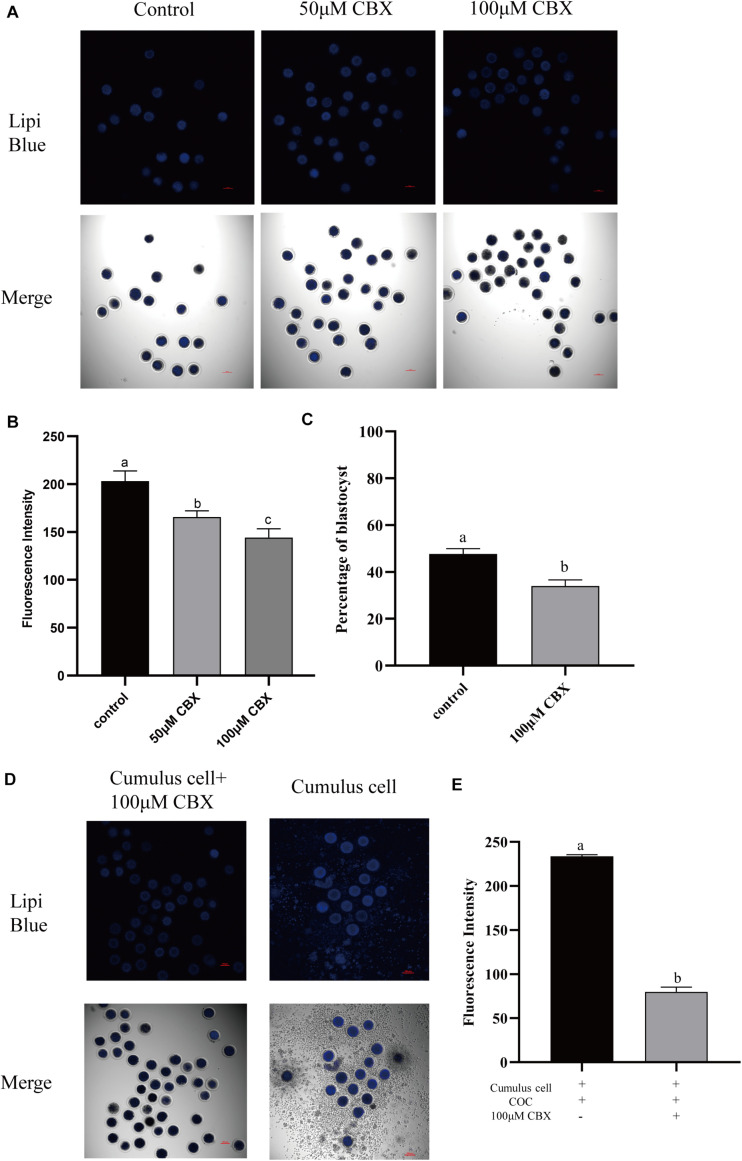
Cumulus cells participate in the regulation of lipid metabolism in porcine oocytes. **(A)** Representative images of Lipi-Blue fluorescence (blue) in porcine oocytes treated with 50 and 100 μM CBX. Scale bar: 100 μm. **(B)** Quantification of fluorescence intensity of Lipi-Blue in porcine oocytes treated with control (*n* = 29), 50 μM CBX (*n* = 28), and 100 μM CBX (*n* = 70). **(C)** The percentage of blastocysts in porcine oocytes treated with control (*n* = 108) and 100 μM CBX (*n* = 44). **(D)** Representative images of Lipi-Blue fluorescence (blue) in porcine oocytes treated with 100 μM CBX and granulosa cell–COC co-cultivation system. Scale bar: 100 μm. **(E)** Quantification of fluorescence intensity of Lipi-Blue in porcine oocytes treated with 100 μM CBX + GC (*n* = 44) and GC (*n* = 16). Student’s *t*-test and one-way ANOVA were utilized for statistical analyses. Different lowercase letters indicate significant differences. Error bars indicate SEM.

During the maturation process, the cumulus cells bidirectionally communicate with oocytes. Here, the transport of lipids from cumulus cells to oocytes was evaluated. Gap junction is pivotal for oocyte–cumulus cell communication. Thus, carbenoxolone (CBX), a gap junction blocker, was supplemented to the maturation medium, and the Lipi-Blue fluorescence intensity in oocytes was significantly deceased at 50 μM (165.73 ± 6.51) and 100 μM (144.30 ± 9.07). Meanwhile, the developmental competence of CBX-treated oocytes was also compromised (33.93 ± 2.7% vs. 47.70 ± 2.3%). When oocytes were co-incubated with cumulus cells which were pre-stained with Lipi-Blue, as shown in Figure 4, the fluorescence intensity (233.60 ± 1.92) was significantly increased. Meanwhile, CBX-treated oocytes showed much lower fluorescence intensity (79.90 ± 5.43). These results indicate that the lipids are transferred from cumulus cells to oocytes through the gap junctions.

### Melatonin Receptors in Cumulus Cells Participate in the Regulation of Lipid Metabolism

**FIGURE 5:**
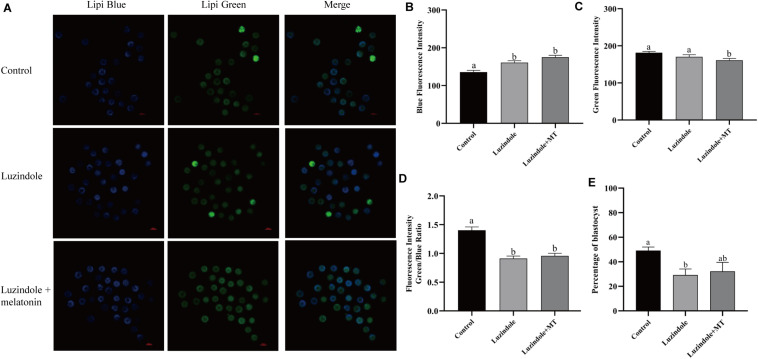
Melatonin receptors participate in the regulation of lipid metabolism in porcine oocytes. **(A)** Representative images of Lipi-Blue fluorescence (blue) in porcine oocytes treated with control, luzindole, and luzindole + melatonin. Scale bar: 100 μm. **(B)** Quantification of fluorescence intensity of Lipi-Blue in porcine oocytes treated with control (*n* = 75), luzindole (*n* = 107), and luzindole + melatonin (*n* = 53). **(C)** Quantification of fluorescence intensity of Lipi-Green in porcine oocytes treated with control (*n* = 70), luzindole (*n* = 94), and luzindole + melatonin (*n* = 57). **(D)** Ratio of fluorescence intensity of Lipi-Blue and Lipi-Green in porcine oocytes treated with control (*n* = 70), luzindole (*n* = 101), luzindole + melatonin (*n* = 52). Student’s *t*-test and one-way ANOVA were utilized for statistical analyses. Different lowercase letters indicate significant differences. Error bars indicate SEM. **(E)** The percentage of blastocysts in porcine oocytes treated with control (*n* = 400), Luzindole (*n* = 204), Luzindole + melatonin (*n* = 203).

**FIGURE 6:**
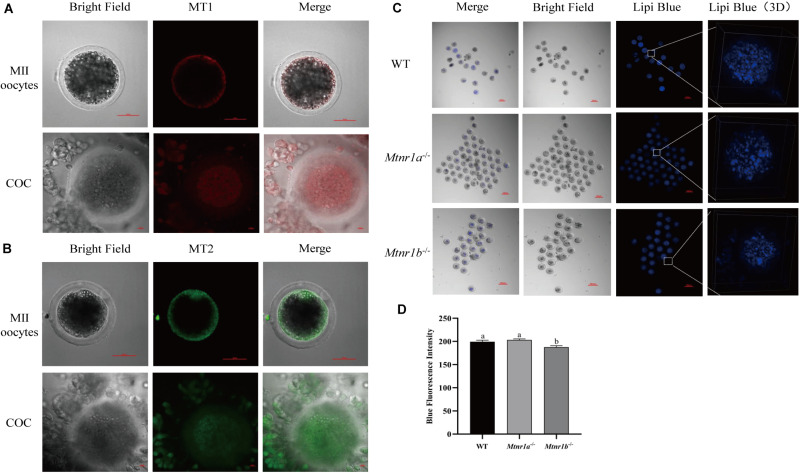
Melatonin receptors express in porcine COCs and participate in the maintenance of lipid metabolism in mouse oocytes. **(A)** The percentage of blastocysts in porcine oocytes treated with control (*n* = 400), luzindole (*n* = 204), and luzindole + melatonin (*n* = 203). **(B)** Representative images of immunofluorescence staining of melatonin receptor 1 (red) and melatonin receptor 2 (green) in porcine oocytes and COC. Scale bar: 100 μm. **(C)** Representative images of Lipi-Blue fluorescence (blue) in C57/BL6 mice MII stage oocytes. Scale bar: 100 μm. **(D)** Representative images of Lipi-Blue fluorescence in WT (*n* = 19), Mtnr1a^–/–^ (*n* = 46), and Mtnr1b^–/–^ (*n* = 23) mice MII stage oocytes. Student’s *t*-test and one-way ANOVA were utilized for statistical analyses. Different lowercase letters indicate significant differences. Error bars indicate SEM.

In order to investigate the mechanism underlying the beneficial effect of melatonin on oocyte maturation, the expression and function of its receptors (MT1/MT2) were evaluated. Firstly, luzindole-treated (the blocker for MT1/MT2) oocytes showed higher blue fluorescent intensity (155.25 ± 5.3), which indicates the green fluorescent intensity was not altered (163.21 ± 6.077) (Figures 5A–D). The treatment with luzindole significantly reduced the porcine oocytes’ ability to develop (29.09 ± 5.0% vs. 49.22 ± 2.9%) (Figure 5E). Meanwhile, immunofluorescence with confocal microscopy observed that both melatonin receptors (MT1/MT2) were expressed in porcine cumulus cells (Figures 6A,B).

As shown in Figures 6C,D, the MII oocytes from melatonin receptor-knockout mice (*Mtnr1a*^–/–^ and *Mtnr1b*^–/–^) were stained with Lipi-Blue, and the fluorescent intensity in oocytes from *Mtnr1b*^–/–^ (187.62 ± 3.02) mice is lower than that from *Mtnr1a*^–/–^ and wild-type mice (203.17 ± 2.3 and 199.31 ± 3.27, respectively). These results indicate melatonin receptors participate in the regulation of lipid genesis and transfer in cumulus cells.

### Melatonin Affects the Gene Expression in Porcine Cumulus Cells

**FIGURE 7:**
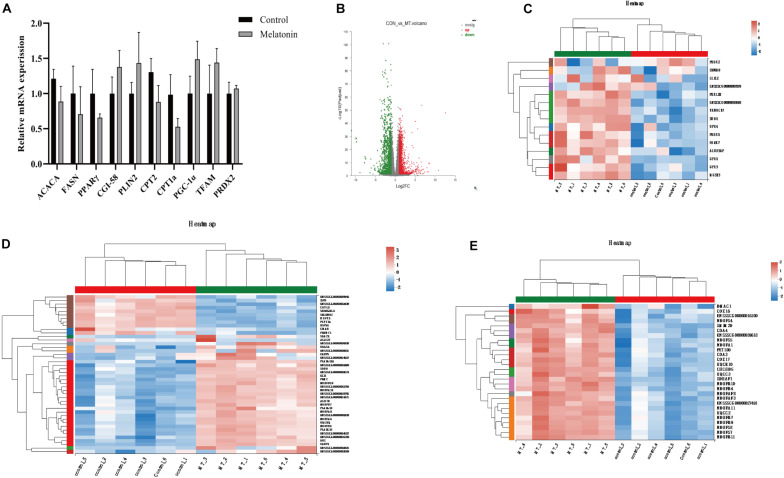
Melatonin affects the gene expression in cumulus cells. **(A)** The mRNA levels of lipid metabolism and mitochondrial biosynthesis in porcine granulosa cells. Different lowercase letters indicate significant differences. Error bars indicate SEM. **(B)** The volcano plot showing upregulated (red) and downregulated (green) gene cumulus cells of the control and melatonin groups. **(C–E)** Heatmap showing average expression for all replicates and relative expression between replicates for genes with antioxidants, lipid metabolism, and mitochondrial respiration chain (based on GO terms).

Transcriptomics analysis of cumulus cells demonstrated that melatonin induces upregulation of 1,528 genes, while 1,193 gene expressions are downregulated (fold change >2 or <0.5) (Figure 7B). As the GO term demonstrated in Figures 7C–E, the top genes altered were related to antioxidants (*SOD1*, *GPX1*, *GPX3*, *GPX4*, *PRDX2*, and *PRDX5*), lipid metabolism (*FABP3*, *FABP5*, *ACACB*, *TECR*, etc.), and mitochondrial respiration (*GPD1*, *ETFB*, *CYC1*, ATP synthases, etc.). Genes related to lipid metabolism and mitochondrial biosynthesis were also evaluated by real-time q-PCR, but no differences were detected between cumulus cells from the control group and those from the melatonin group (Figure 7A).

## Discussion

Mammalian oocytes consume energy to achieve their final maturation such as germinal vesicle breakdown (GVBD), extrusion polar body, and redistribution of organelles. The basic pattern of energy production and utilization in mammalian oocytes has been recognized for more than half a century (Leese et al., 2016). It has been demonstrated that porcine oocytes are able to consume glucose and pyruvate to support their final maturation. However, in recent years, evidence that lipids are a pivotal nutrient for porcine or even the main energy source has accumulated (Bradley and Swann, 2019). In the current research, methods to monitor the dynamics of lipids in maturing porcine oocytes were established with Lipi-Blue and Lipi-Green probes. Together with lipid metabolism-related agonists, melatonin was found to suppress lipolysis but to increase lipid accumulation in porcine oocytes. Cumulus cells and melatonin receptors play a critical role in regulating oocyte lipid store for both pig and mouse, and melatonin targets the cumulus cells to promote oocyte maturation.

Porcine oocytes store exceptionally high amounts of endogenous lipid when compared with those in all other mammalian oocytes (McEvoy et al., 2000). In recent years, great progress had been made in elucidating the role of lipid metabolism in energy generation during oocyte maturation and early embryo development. Now, the morphological and biochemical changes in LDs can be considered important indicators of successful resumption of meiosis and maturation accomplishment. Here, we apply Lipi-Blue and Lipi-Green to detect the dynamics of LDs in live single oocytes. These probes are LD-specific fluorescent probes which allow monitoring of LDs in live cells even 48 h after staining and overcome the drawbacks of traditional dyes such as Nile Red and BODIPY 493/503 (Tatenaka et al., 2019). We successfully monitored the changes of lipids in live single porcine oocytes. The results confirmed that the lipid contents decreased as the oocytes went through the final maturation. Based on the decomposition of Nile Red spectra, researchers detected significant decreases in the level of triglycerides (17.7%), phospholipids (26.4%), and cholesterols (23.9%) in porcine oocytes from immature to mature stages (Romek et al., 2011).

Meanwhile, previous studies found that oocytes with LDs centrally located showed a significantly higher rate of blastocyst development than the oocytes with LDs localized uniformly in the whole cytoplasm (Hiraga et al., 2013). In the current study, we observed that the LDs were accumulated in the center of porcine oocytes and that the treatment of etomoxir abolished the redistribution of LDs. Melatonin cannot correct the abnormal distribution of LDs. We used UPLC–MS/MS to analyze biochemical composition changes in oocytes. Another study using DESI–MS described differences in lipid composition among immature and *in vitro* mature porcine oocytes (Pirro et al., 2014). The disruption of the beta-oxidation pathway in mouse and cattle oocytes was found to negatively affect nuclear maturation and subsequent developmental potential (Ferguson and Leese, 2006; Dunning et al., 2010), and stimulation of FAO in mouse oocytes enhanced nuclear maturation (Dunning et al., 2011; Paczkowski et al., 2014). But the stimulation of lipolysis leads to inconsistent outcomes of oocyte nuclear and cytoplasmic maturation (Lowe et al., 2019). It was found that that stimulation of lipid metabolism by L-carnitine is beneficial to meiotic progression for porcine oocytes (Somfai et al., 2011; Wu et al., 2011), whereas another study showed that L-carnitine does not affect nuclear maturation (You et al., 2012). With the probes, it was observed that the disruption of lipolysis, lipid genesis, or mitochondrial activity all cause defects in oocyte maturation and development. It is evidenced that lipid metabolism is active throughout oocyte maturation and that melatonin modulates lipolysis and biogenesis to balance the energy supply and protect oocytes from ROS.

The beneficial effects of melatonin on mammalian oocytes have been well recognized. Evidence that melatonin enhances porcine oocyte maturation and embryo development has been accumulated, but the detailed mechanism is still unrevealed. Here, we present evidence to distinguish whether melatonin promotes porcine oocyte maturation through its receptors or its anti-oxidation capability. We observed that melatonin slows down the mobilization of lipids stored in the oocytes, indicated by a lower fluorescence intensity at the MII stage than at the GV stage. In a previous study, it was claimed that oocytes treated with melatonin formed smaller LDs and increased the content of FAs, mitochondria number, and ATP level (Jin et al., 2017). But here, we found that melatonin suppresses lipolysis by evaluating the fluorescence intensity remaining in MII oocytes. However, another study showed that melatonin benefits porcine oocyte maturation by enhancing LD accumulation and triglyceride content in porcine oocytes (He et al., 2018). As we have observed, melatonin enhances the accumulation of lipids in oocytes. Together with the two previous researches, it can be speculated that melatonin balanced lipid biogenesis and utilization for the oocytes to generate sufficient ATP and limited ROS production to support the maturation of oocytes and subsequent embryonic development. Due to the lack of methods to observe ATP and ROS in live single cells, we are still not able to quantify the proper level of ATP/ROS for oocyte IVM.

Porcine oocytes are unable to accomplish their final maturation without the cumulus cell layer. Normally, the layers of cumulus cells surrounding ovulated oocytes directly have contact with the follicular fluid and bidirectionally transport small metabolites to regulate the growth and maturation of oocytes through gap junctions (Prates et al., 2014). In the current study, when the gap junction was disrupted by CBX, the oocytes showed lower amounts of lipids and compromised developmental competence. The cumulus cell layers play an important role in regulating lipid metabolism and transmitting the changes of free FA levels in the follicular fluid or culture medium. It had been observed that the cumulus cells functionally affect cytoplasmic mitochondria-lipid distributions in porcine oocytes that matured *in vitro* and determine the acquisition of developmental competence (Cui et al., 2009). The latest research found that cumulus cells may balance FA accumulation in porcine oocytes, thus securing oocyte maturation. The supplementation of stearic acid in the maturation medium significantly increases the size and number of LDs in the cumulus cells but not those in oocytes; thus, it did not significantly alter the subsequent embryo development (Pawlak et al., 2020). When bovine oocytes are exposed to unsaturated or saturated FAs, the cumulus cells can store elevated FA in either the culture medium or follicle fluid to keep the ratio of saturated and unsaturated FAs in the proper range, thus protecting maturing oocytes from lipotoxicity (Aardema et al., 2011, 2013). The evidence presented here showed that cumulus cells directly participate in the regulation of the lipid store of oocytes. Blocking the gap junction could reduce the transport of lipids from cumulus cells to oocytes.

As we previously reported, melatonin receptors were identified on cumulus cells and oocytes of sheep and bovine (Tian et al., 2014, 2017; Wang et al., 2014). But for porcine oocytes, only RT-PCR analysis revealed the expression of the MT1 gene in cumulus and granulosa cells but not in oocytes (Kang et al., 2009). The MT2 receptor mediated the stimulatory effects of melatonin on porcine cumulus expansion and subsequent embryo development (Lee et al., 2018). MT1 and MT2 were expressed in porcine granulosa cells (He et al., 2016). No research had been performed to investigate the role of melatonin on metabolism in cumulus cells. We found that the inhibitors of melatonin receptors remarkably reduced lipid mobilization and lipid biogenesis. Meanwhile, melatonin affects the expression of genes related to antioxidants, lipid metabolism, and mitochondrial respiration in cumulus cells. Altogether, melatonin receptors in cumulus cells play an essential role in conducting the regulatory signal induced by melatonin.

Our results provide a new perspective that reveals the mechanism of the beneficial effects of melatonin on porcine oocytes. With the methods to monitor lipid dynamics in maturing oocytes, it was confirmed that the lipid content decreases as maturation progresses. Melatonin may play an important role in balancing lipid biogenesis and utilization. It was observed that lipids are transferred from cumulus cells to oocytes through gap junctions and that melatonin receptors participate in this process. In summary, melatonin promotes oocyte maturation by regulating lipid metabolism through its receptors in cumulus cells. In the future, our results may be applied to clinical settings or the livestock industry where melatonin will be supplied to the diet for sows undergoing estrus or female undergoing ART treatment to help them generate high-quality oocytes.

## Data Availability Statement

The datasets presented in this study can be found in online repositories. The names of the repository/repositories and accession number(s) can be found in the article/supplementary material. The datasets generated for this study can be found in NCBI BioProject Accession ID PRJNA703733.

## Ethics Statement

The animal study was reviewed and approved by the Animal Care and Use Committee of China Agricultural University.

## Author Contributions

LZ and GL proposed the research and revised the manuscript. TZ, SG, DL, MZ, LY, LS, and PJ performed the experiments. TZ and LZ analyzed the data and drafted the manuscript. All authors contributed to the article and approved the submitted version.

## Conflict of Interest

The authors declare that the research was conducted in the absence of any commercial or financial relationships that could be construed as a potential conflict of interest.

## Footnotes

1 http://www.hmdb.ca/

2 https://metlin.scripps.edu/

3 https://github.com/jstjohn/SeqPrep

4 https://github.com/najoshi/sickle

5 http://asia.ensembl.org/Sus_scrofa/Info/Index

6 http://tophat.cbcb.umd.edu/

7 http://deweylab.biostat.wisc.edu/rsem/

8 http://www.bioconductor.org/packages/2.12/bioc/html/edgeR.html

9 https://github.com/tanghaibao/Goatools

10 http://kobas.cbi.pku.edu.cn/home.do

11 http://bioconductor.org/packages/release/bioc/html/ropls.html

## Abbreviations

LDs: Lipid droplets
CPT: Carnitine palmitoyl transferase
ACSLs: Long-chain acyl-coa synthetases
MT1: Melatonin receptor 1
MT2: Melatonin receptor 2
mtDNA: Mitochondrial DNA
DNMT1: DNA methyltransferase 1
COCs: Cumulus–oocytes complexes
ETC: Electron transport chain
DEGs: Differential expression genes
FRKM: Fragments per kilobase of exon per million mapped reads
PCA: Principle component analysis
GV: Germinal vesicle
GVBD: Germinal vesicle breakdown
FAO: Fatty acid oxidation
CBX: Carbenoxolone
ROS: Reactive oxygen species.
