# Corrigendum: Melatonin Modulates Lipid Metabolism in Porcine Cumulus–Oocyte Complex via Its Receptors

**DOI:** 10.3389/fcell.2021.727612

**Published:** 2021-07-12

**Authors:** Tianqi Zhu, Shengyu Guan, Dongying Lv, Mengmeng Zhao, Laiqing Yan, Li Shi, Pengyun Ji, Lu Zhang, Guoshi Liu

**Affiliations:** Key Laboratory of Animal Genetics, Breeding and Reproduction, National Engineering Laboratory for Animal Breeding, Key Laboratory of Animal Genetics Improvement, Ministry of Agriculture, College of Animal Science and Technology, China Agricultural University, Beijing, China

**Keywords:** melatonin, cumulus cell, oocyte, lipid metabolism, melatonin receptor

In the published article, there was an error in affiliation 1, 2. Instead of “^1^Key Laboratory of Animal Genetics, Breeding and Reproduction, National Engineering Laboratory for Animal Breeding, Key Laboratory of Animal Genetics Improvement, Ministry of Agriculture, Beijing, China. ^2^College of Animal Science and Technology, China Agricultural University, Beijing, China,” it should be “Key Laboratory of Animal Genetics, Breeding and Reproduction, National Engineering Laboratory for Animal Breeding, Key Laboratory of Animal Genetics Improvement, Ministry of Agriculture, College of Animal Science and Technology, China Agricultural University, Beijing, China.”

The authors apologize for this error and state that this does not change the scientific conclusions of the article in any way. The original article has been updated.

